# Techno-economic viability of natural hydrogen

**DOI:** 10.1093/nsr/nwaf368

**Published:** 2025-09-03

**Authors:** Kaiqiang Zhang, Xingjie Ma, Boyue Zheng, Zhijun Jin

**Affiliations:** Institute of Energy, Peking University, China; School of Earth and Space Sciences, Peking University, China; Institute of Carbon Neutrality, Peking University, China; Ordos Research Institute of Energy, Peking University, China; Institute of Energy, Peking University, China; School of Earth and Space Sciences, Peking University, China; Institute of Energy, Peking University, China; School of Earth and Space Sciences, Peking University, China; Institute of Energy, Peking University, China; School of Earth and Space Sciences, Peking University, China; Institute of Carbon Neutrality, Peking University, China; Ordos Research Institute of Energy, Peking University, China

## Abstract

This study conducted a comprehensive economic assessment of natural hydrogen extraction scenarios, evaluating techniques using indicators such as unit cost, net present value, and payback period. By analyzing 32,000 scenarios with varying hydrogen purity, volumes, well depths, separation techniques, production well numbers, and off-gas disposal well numbers, we aim to provide essential theoretical guidance and practical insights to reduce production costs.

Hydrogen is regarded as an important future energy source, supporting the net-zero transition [1]. In recent years, its application has expanded across transportation energy terminals and other sectors, driven by emerging technologies such as hydrogen fuel cells [2]. However, conventional hydrogen production methods either result in high greenhouse gas emissions [3] or require the integration of carbon capture and storage (CCS) technologies, which significantly increase production costs [4]. While hydrogen produced from renewable energy sources is carbon-free, the associated high cost remains a major barrier to widespread commercial adoption [5]. By contrast, natural hydrogen offers dual advantages of zero greenhouse gas (GHG) emissions and potentially lower production costs compared to anthropogenic hydrogen.

The identification of natural hydrogen reserves has sparked increasing global exploration [6,7] and research into this valuable resource, with the commercialization of natural hydrogen now gaining momentum [8]. Natural hydrogen resources are currently recognized in two primary forms: gas reservoir resources (free gas accumulations) and dissolved gas resources (hydrogen dissolved in aquifers). This distinction is crucial as it fundamentally influences extraction parameters, applicable technologies and economic viability. While the exploitation of gas reservoir resources is already underway, the development of technologies for extracting dissolved gas resources remains in its early stages, with limited documented data available. Although several in-depth investigations

have been conducted into various aspects of natural hydrogen reserves, including geological discovery [9], formation mechanisms [10] and GHG emissions during production [11], there remains a lack of comprehensive studies assessing the economic and technological feasibility of natural hydrogen extraction. This research therefore explores the complex technical methods involved in producing natural hydrogen, focusing on gas reservoir–type resources and taking into account both natural conditions and engineering factors. Key economic indicators such as unit cost, net present value (NPV) and payback period are also analyzed. Through this comprehensive economic and technical assessment, this study aims to provide essential theoretical guidance and practical insights for the extraction of natural hydrogen in current and future applications.

In the natural hydrogen extraction process, six major parameters are considered: hydrogen purity, hydrogen volume, well depth, separation technique, number of production wells and number of off-gas disposal wells. These parameters varied across eight levels of hydrogen purity, five levels of hydrogen volume, five well depths, four separation techniques, eight production well configurations and five off-gas disposal well configurations, resulting in a total of 32 000 scenarios (Fig. 1b). All scenarios were analyzed to determine the range of unit costs for natural hydrogen production, which was found to be between $0.14 and $5.33 per kilogram of H_2_ (for data analysis see the Excel file in Supplementary Data online). Subsequently, a detailed analysis was

conducted to examine the trend in unit cost in relation to hydrogen purity and volume across the four separation techniques, under fixed conditions (i.e. 400 m well depth, 20 production wells and one off-gas disposal well) (Supplementary Text S6).

**Figure 1.**
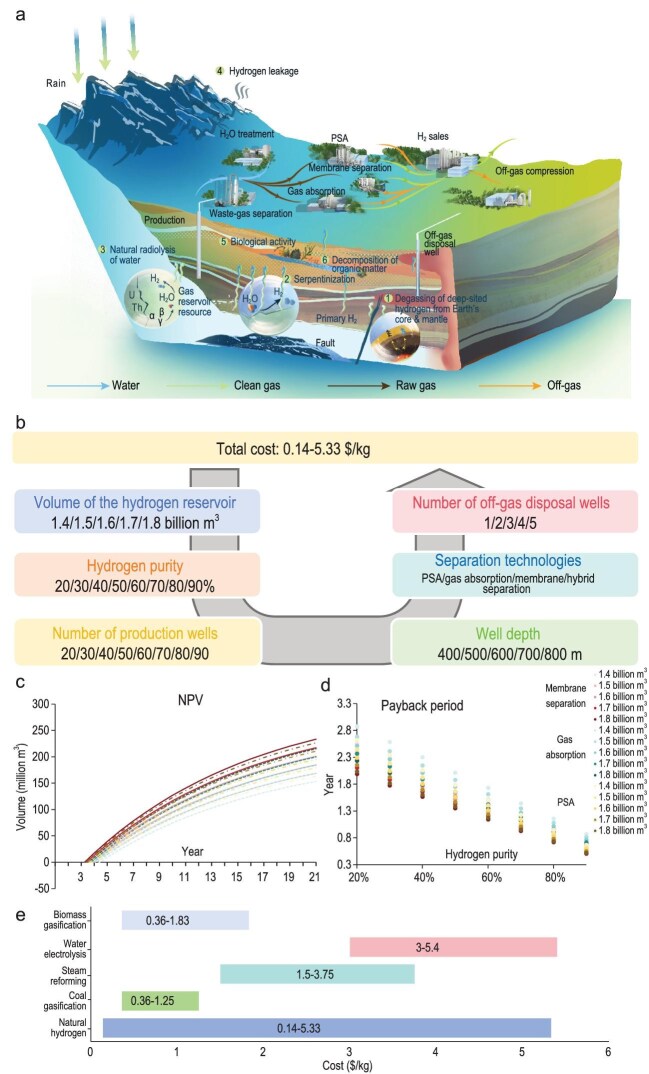
(a) The extraction process of natural hydrogen (1. Degassing of deep-seated hydrogen from the Earth’s core and mantle. 2. Serpentinization is a metamorphic process in which mainly ultrabasic rocks are oxidized by water into serpentine producing hydrogen. 3. The Earth’s crust contains significant quantities of radioactive elements mainly U, Th and K, and the energy from radioactive decay is high enough to break apart molecules of water into their component parts, oxygen and hydrogen. 4. Hydrogen leakage 5. Hydrogen can be produced biologically via the anaerobic decay of organic matter, fermentation and by nitrogen fixing bacteria. 6. Decay of organic matter with hydrogen as the result). (b) Technical and economic database of natural hydrogen extraction. (c) The NPV of natural hydrogen extraction. (d) The payback period of natural hydrogen extraction. (e) Cost comparison for different hydrogen production technologies.

When evaluating NPV, as detailed in Supplementary Text S5, eight levels of hydrogen purity, five hydrogen volumes and three separation techniques were considered. Due to the extensive number of potential scenarios, the analysis was conducted under the assumption of a constant well depth of 500 m, with 20 production wells and one off-gas disposal well. These parameters were selected based on the scenarios presented in Fig. 1c, with a focus on 15 scenarios at 90% H_2_ purity (NPV curves for different purities are shown in Supplementary Text S2). As illustrated in Fig. 1c, the NPV curve demonstrates the following characteristics: NPV is negative in the first year due to the aggregation of all capital cost components. From the second year onwards, NPV for all scenarios increases and transitions into a positive value. The rate of NPV growth gradually declines over time, and the trend varies depending on the separation technique employed. The highest rate of change is observed when pressure swing adsorption (PSA) is used, in combination with a hydrogen volume of 1.8 billion m^3^, compared to other separation methods. From this analysis, it can be concluded that NPV becomes positive in the second year and continues to grow in subsequent years, indicating that investment in natural hydrogen extraction has a strong potential for economic viability.

The payback period reflects the speed of capital recovery. The selection of scenarios for calculating the payback period follows a similar methodology to that used for NPV analysis, with the exception that the number of production wells is determined based on hydrogen purity (120 scenarios are illustrated in Fig. 1d). In particular, for natural hydrogen with purities ranging from 20% to 90%, the corresponding numbers of production wells are 90, 80, 70, 60, 50, 40, 30 and 20, respectively. Across these scenarios, payback periods range from 0.5 to 2.9 years. A clear trend is observed in which the payback period value increases as hydrogen purity decreases, despite the total hydrogen volume remaining constant. In all analyzed scenarios, the payback period is significantly shorter than the 20-year operational lifespan of the system. This suggests that investment in natural hydrogen extraction is economically viable and entails relatively low financial risk due to a rapid capital turnover.

A comparison between natural hydrogen and other hydrogen production technologies was also conducted (Fig. 1e). Gray and Tomlinson conducted a cost analysis of hydrogen production via coal gasification under three scenarios, reporting a cost range between $0.36 and $1.25 per kilogram of H_2_ [12]. Williams *et al.* [13] reported that hydrogen produced via steam reforming of natural gas costs between $1.50 and $3.75 per kilogram. When CCS is integrated with these methods, the production costs increase marginally. By comparison, natural hydrogen extraction at higher purities in this study shows a significantly lower cost, reaching as low as $0.14 per kilogram of H_2_. Even compared to large-scale coal gasification without CCS ($0.36 per kilogram of H_2_), natural hydrogen retains a notable competitive advantage. In fact, it can be up to ten times more cost-effective than the average cost of conventional hydrogen production technologies. Specifically, when comparing the average costs, natural hydrogen extraction is ∼61.1% less expensive than conventional methods. For renewable hydrogen production, Lemus and Martínez Duart [14] proposed nuclear-powered electrolysis, with an estimated cost between $3.00 and $5.40 per kilogram of H_2_. Additionally, Yukesh Kannah *et al.* [15] reported that the cost of hydrogen from biomass gasification ranges between $0.36 and $1.83 per kilogram. Although renewable hydrogen technologies yield zero-carbon emissions, they tend to be more costly than fossil fuel–based methods. From the comparative analysis, it is evident that natural hydrogen offers a significant cost advantage, particularly when its purity exceeds 60%, with production costs ranging from $0.14 to $3.05 per kilogram. This purity threshold effectively serves as a benchmark for assessing the economic viability of natural hydrogen extraction.

In summary, this study encompasses a total of 32 000 scenarios, revealing that the unit cost of natural hydrogen production ranges from $0.14 to $5.33 per kilogram of H_2_. Comparative analysis with other hydrogen production techniques highlights the significant cost advantages associated with extracting high-purity natural hydrogen. Furthermore, across the 120 selected scenarios, evaluations of NPV and payback period indicate that projected financial returns outweigh investment costs, thereby affirming the economic viability of the project. Based on this comprehensive assessment, the extraction of natural hydrogen with a purity exceeding 60% demonstrates considerable economic potential, with cost competitiveness reaching up to $5.26 per kilogram of H_2_.

## Supplementary Material

nwaf368_Supplemental_Files

## Notes

***Conflict of*  *interest statement.*** None declared.
