# Supplementary material for: Techno-economic viability of natural hydrogen
Source: Natl Sci Rev. 2025 Sep 3;12(10):nwaf368. doi: 10.1093/nsr/nwaf368 (PMC12485604; doi:10.1093/nsr/nwaf368)
Supplement: nwaf368_Supplemental_Files [file nwaf368_supplemental_files.zip › SI-text-Natural Hydrogen.docx]

Supplemental material**s**

**Techno-Economic Viability of Natural Hydrogen**

Kaiqiang Zhang^1,2,3,4^*, Xingjie Ma^1,2^, Boyue Zheng^1,2^, Zhijun Jin^1,2,3,4^

^1^Institute of Energy, Peking University, Beijing, People's Republic of China

^2^School of Earth and Space Sciences, Peking University, Beijing, People's Republic of China

^3^Institute of Carbon Neutrality, Peking University, Beijing, People's Republic of China

^4^Ordos Research Institute of Energy, Peking University, Ordos, People's Republic of China

Corresponding Authors:

Kaiqiang Zhang

Institute of Energy, Peking University, Beijing 100871, China

Email: kaiqiang.zhang@pku.edu.cn

[S1: Parameters of natural hydrogen reservoirs 3](#_Toc21197)

[hydrogen purity 3](#_Toc30319)

[hydrogen volume 3](#_Toc15449)

[S2: Establishment and cost data of natural hydrogen extraction procedure 3](#_Toc3544)

[wells drilling 3](#_Toc20121)

[separation techniques 4](#_Toc20425)

[S3: Methods for NPV, P and unit cost 5](#_Toc6226)

[S4: Assumptions and data tables for scenario 6](#_Toc22304)

[S5: NPV and P at different hydrogen purity 6](#_Toc7016)

[NPV at different hydrogen purity 6](#_Toc11942)

[Parameters of payback period 8](#_Toc28182)

[S6: Unit costs of natural hydrogen extraction for various hydrogen volumes, purity and separation techniques 8](#_Toc14333)

# S1: Parameters of natural hydrogen reservoirs

## hydrogen purity

The economic performance of natural hydrogen extraction is largely influenced by the variability in hydrogen purity. Investigation of the hydrogen purity facilitates detailed analysis of economic implications. Currently, a comprehensive review for hydrogen purity is conducted by Zgonnik (*1*),which included 100s of gas samples wherein hydrogen purity greater than 10%:

- Samples with greater than 90% hydrogen have been discovered in many countries such as

Oman, USA, Australian, Japan, Russian Federation, Uzbekistan and Belans.

- Samples with greater than 90% hydrogen have been observed in types of samples including

Ophiolites, rift zones, precambrian rocks, volcanic gases, orebodies, coal basins,

sedimentary basins, and salt deposits.

- The relatively low purity hydrogen has been seen in more countries and types of deposits.

Based on the above review, we set the natural hydrogen purity at 20%, 30%, 40%, 50%, 60%, 70%, 80%, 90% for economic analysis.

## hydrogen volume

The volume of natural hydrogen is also a crucial element influencing the economic performance. Adam R. Brandt (*2*) investigated the volume of natural hydrogen, which is 57 billion cubic feet (1.61 Bm^3^). Considering the diverse volumes of natural hydrogen reservoirs, we employ these variations as a basis for subsequent considerations including 1.4, 1.5 1.6, 1.7, 1.8 Bm^3^.

# S2: Establishment and cost data of natural hydrogen extraction procedure

## wells drilling

In order to estimate the depth range for hydrogen extraction wells, we conducted research on the depths of existing hydrogen wells. Data for well depth is derived from Alain Prinzhofer (*3*) and J. Guelard (*4*), including wells in Kansas and Mali:

- The studied wells located in Kansas are Heins#1, Scott#1, and Sue Duroche#2. The Heins #1 well was drilled in 1981 and is located in Morris County with a depth of 770 m. The Scott#1 well was drilled in 1982 and is located in Geary County a few kilometers southeast of Sue Duroche#2 well with a depth of 677 m. The Sue Duroche#2 well was drilled in 2008 with a depth of 424 m.
- Wells in Mali including the Bourakebougou pioneer well (1987) and 2018 exploratory wells. The Bourakebougou water well (Bougou-1), was cemented after a gas explosion occurred during drilling operations. The incident occurred when a measured drilled depth of 112 m was reached. The exploratory wells are located within a radius of 8 km and the total depth for the different wells ranges from 105 m to 1807.4 m.

Based on the review of well depth, the depth of 400, 500, 600, 700, 800m are taken into account.

The number of producer and off-gas disposal wells is referenced to the natural hydrogen extraction procedure set up by Adam R. Brandt(*2*). On this basis, we expand the producer wells (i.e., 20, 30, 40, 50, 60, 70, 80) and off-gas disposal wells (i.e., 1, 2, 3, 4, 5).

Cost data for wells drilling is derived from Lukawski et al.’s research (*5*), which is a cost analysis of oil, gas, and geothermal well drilling. The research encompassed the average depth of onshore oil and gas well in U.S. and cost data are obtained from API JAS. Data include both vertical and directional wells. Also, we make assumption that the natural hydrogen fields exhibit similarities to gas wells. Based on the above, we get the following summary cost data shown in Table S1 below.

**Table S1: cost data of wells drilling for analysis**

| **Depth interval(m)** | **Value($/m)** |
| --- | --- |
| 381-761 | 1263.12 |
| 762-1142 | 1056.43 |
| 1143-1523 | 885.83 |
| 1524-2285 | 1437.01 |

## separation techniques

The procedure for separation techniques is adapted from hydrogen recovery processes utilized in the Tehran refinery off-gas system(*6*), including pressure-swing absorption(PSA), membrane separation and gas absorption (Figure S1(a), (b), (c)):

- The PSA process relies on absorption mechanisms, employing adsorbents such as molecular sieve, activated carbon, and silica gel are used in PSA beds. Activated carbon and silica gel are used for purified hydrogen. Typically, multiple beds are needed in process for production of purified hydrogen, while one of them is producing high purity product, other beds are being regenerated at the same time.
- Hydrogen purification via membrane separation relies on disparities in the permeability of components within the feed stream. Gases with higher permeability traverse the membrane more rapidly than others. In this process, hydrogen exhibits greater permeability compared to methane and heavier hydrocarbons, allowing it to pass through the membrane at an accelerated rate. Consequently, the product stream becomes enriched in hydrogen.
- Gas absorption, specifically the absorption of light hydrocarbons from a hydrogen stream into a hydrocarbon solvent, is commonly referred to as the sponge oil process. This method relies on the solubility of hydrocarbons in the chosen solvent. In this process, pure hydrogen is generated by passing the feed stream through an absorption tower, where impurities are absorbed by solvent at feed pressure and temperature below 0 ℃.

When dealing with low-purity natural hydrogen, hybrid separation techniques become necessary to obtain high-quality product hydrogen (Figure S1(d)). The development of procedures is informed by hybrid technologies designed for separating and purifying hydrogen from natural gas grids, utilizing novel membrane-based hybrid technology.


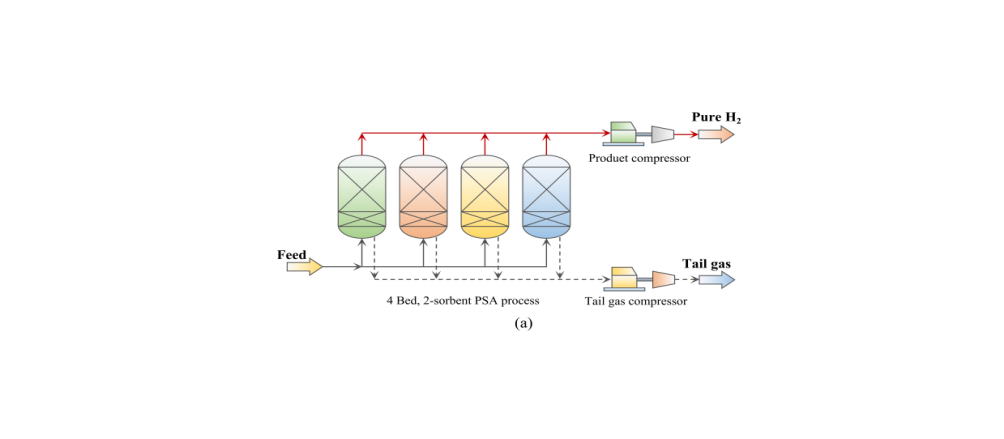

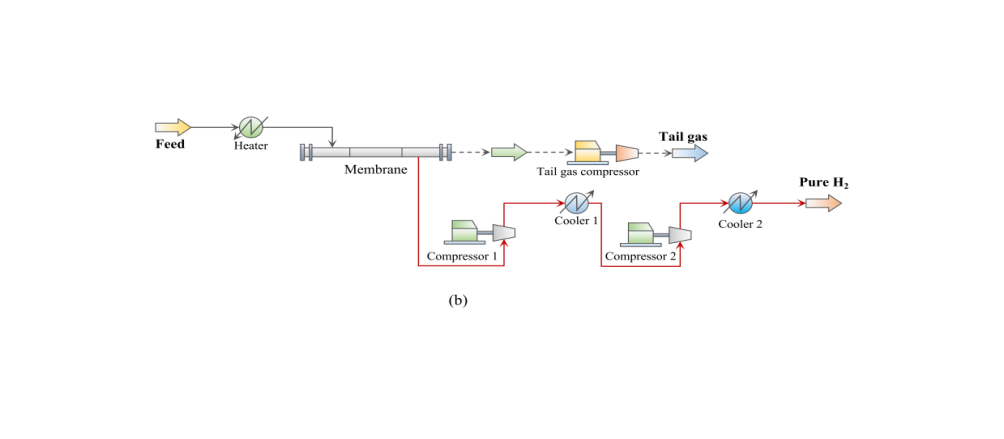

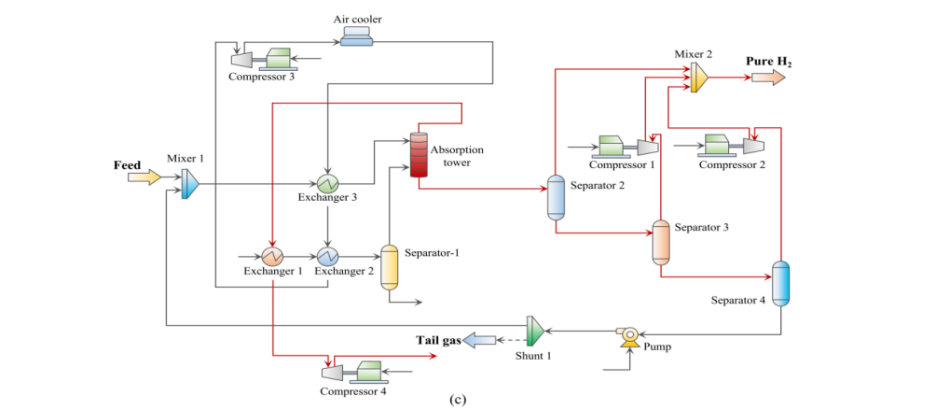

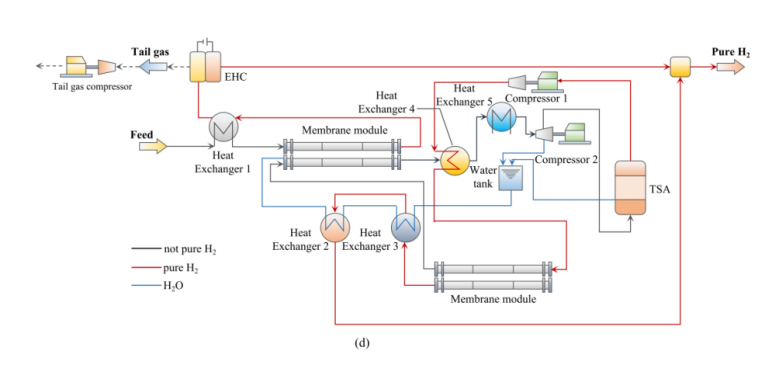


Figure S1 Flow diagram of separation techniques for nature hydrogen purification (a) PSA separation (b) membrane separation (c) gas absorption (d) hybrid separation (combining novel membrane-based hybrid technologies)

# S3: Methods for NPV, P and unit cost

The unit cost of nature hydrogen produced is calculated through the ratio between the sum of the capital (CAPEX) and operational costs (OPEX) and the hydrogen flow rate mined, shown in Eq. (1).

$unit cost of nature hydrogen=\frac{OPEX\times PLT+CAPEX}{m_{H_{2}}\times PLT}$ Eq. (1)

where PLT is the project lifetime, m_H2_ is hydrogen production quality per year.

The NPV of an investment refers to the process of discounting the future cash flows to their present value at the initial investment date, which depends on the discount rate, investment value and lifetime. It is a dynamic evaluation index that reflects the profitability of investment program in the calculation period and provides a method for evaluating and comparing capital projects or [financial products](https://en.wikipedia.org/wiki/Financial_product) with cash flows spread over time. NPV is defined as the sum of the present values of all the costs and benefits incurred by the project as reported in Eq. (2).

$NPV(X,r)=\sum_{t=0}^{\mathrm{PLT}} \frac{{(B}_{t}-C_{t})}{{(1+r)}^{t}}$ Eq. (2)

where B_t_ is the benefits in the year t, C_t_ is the costs in the year t, r is the discount rate, t is the occurring year.

The payback time is the period, which the initial investment is completely recovered, as expressed in Eq.(3).

$P=\frac{\mathrm{CAPEX}}{b-c}$ Eq. (3)

where P is the payback period, b is the projected annual benefits, c is the projected annual operating costs.

# S4: Assumptions and data tables for scenario

**Table S2: natural hydrogen extraction parameters for analysis**

| **Parameter** | **Value** | **Unit** |
| --- | --- | --- |
| system operational cycle | 20 | year |
| H_2_ purity | 20, 30, 40, 50, 60, 70, 80, 90 | mol% |
| H_2_ volume | 1.4, 1.5,1.6, 1.7, 1.8 | Bm^3^ |
| well depth | 400, 500, 600, 700, 800 | m |
| number of production wells | 20, 30, 40, 50, 60, 70, 80, 90 | wells |
| number of off-gas disposal wells | 1, 2, 3, 4, 5 | wells |
| pressure drop | 0 | % per year |
| production decline rate | 0 | % per year |

**scenarios setting**

- We consider total 32000 scenarios to determine the range of unit costs, due to 8 hydrogen purity, 5 hydrogen volumes, 5 well depths, 8 numbers of production wells, 4 separation techniques and 5 numbers of off-gas disposal wells.
- In order to be closer to actual production, for NPV and P, we fix well depth, number of production wells and off-gas disposal wells. Meanwhile, we only consider 3 lower cost separation techniques including PSA, membrane separation and gas absorption, thus a total of 120 scenarios are taken into account in these two parts.

# S5: NPV and P at different hydrogen purity

## NPV at different hydrogen purity

As shown in methods, NPV value is related to many indicators. For a specific purity of natural hydrogen, the primary variables include hydrogen volume and separation technology, resulting in a total of 15 potential scenarios. We consider NPV values at more natural hydrogen purity, as shown in Figure S2. (i.e., 500 well depth, 20 producer wells, one off-gas disposal well)


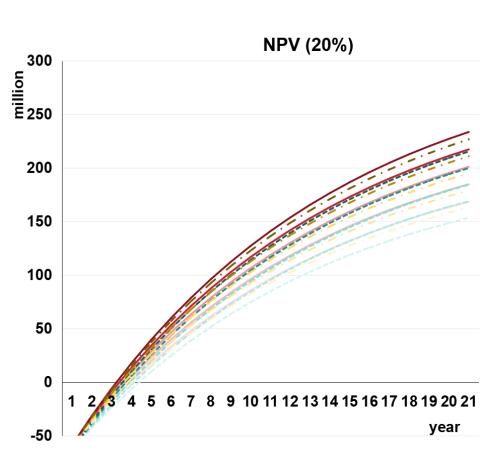

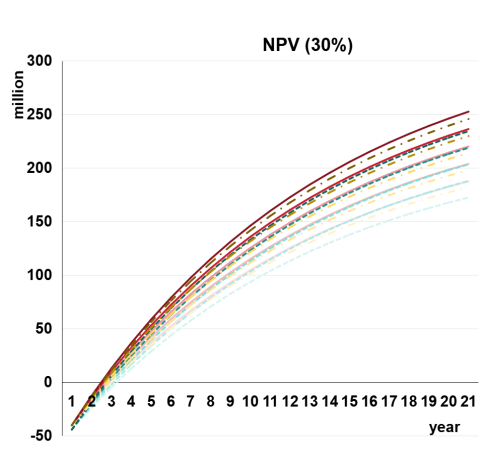

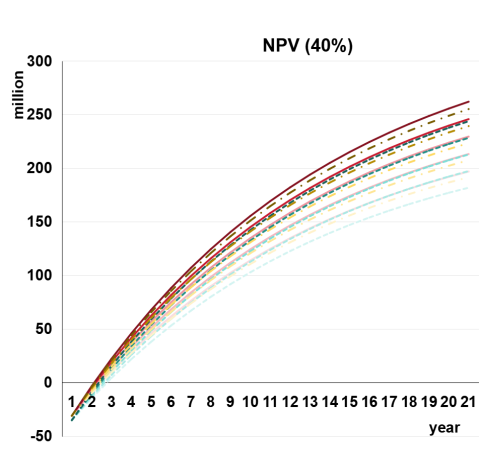

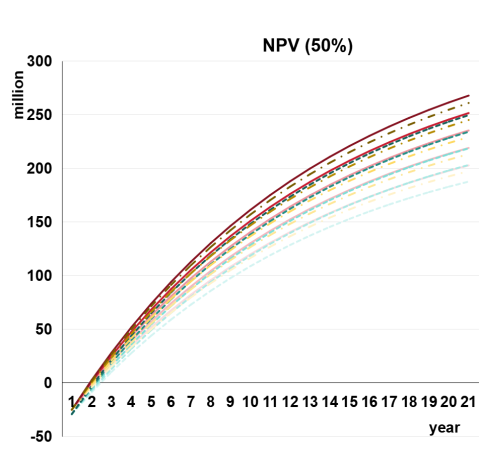

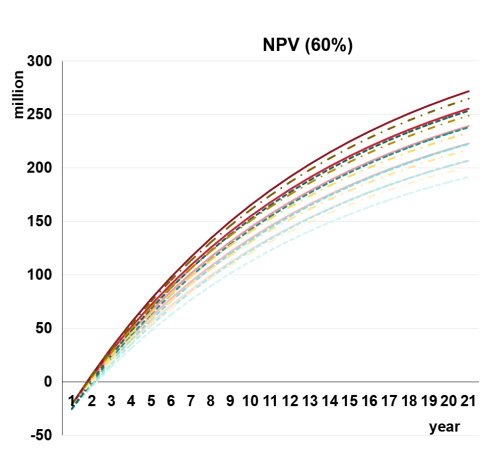

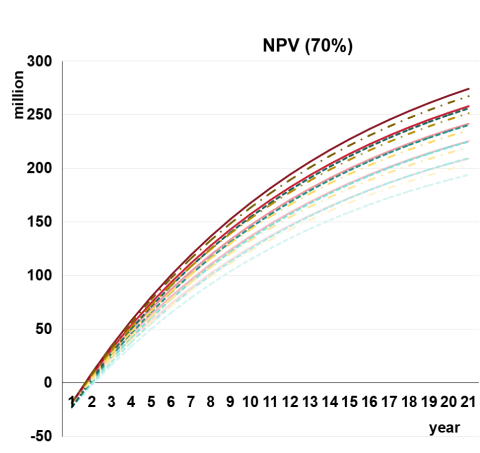

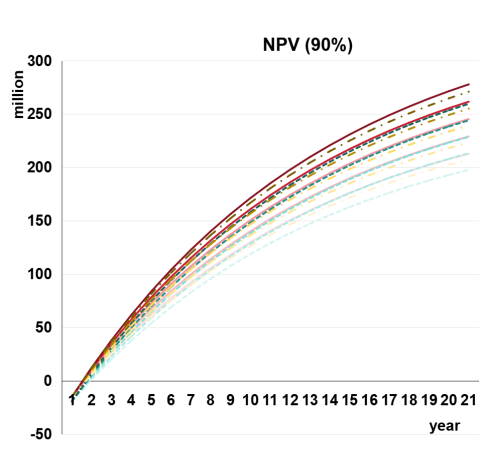

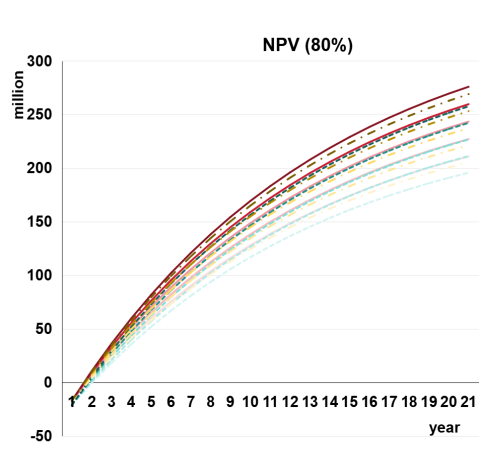

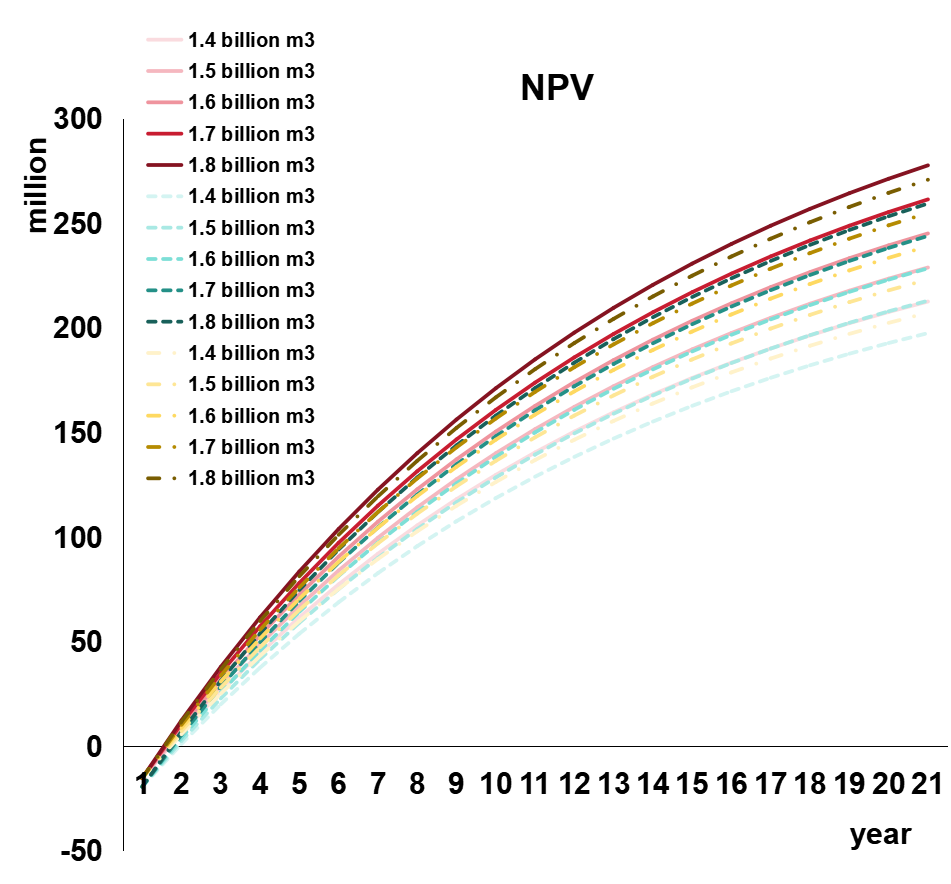

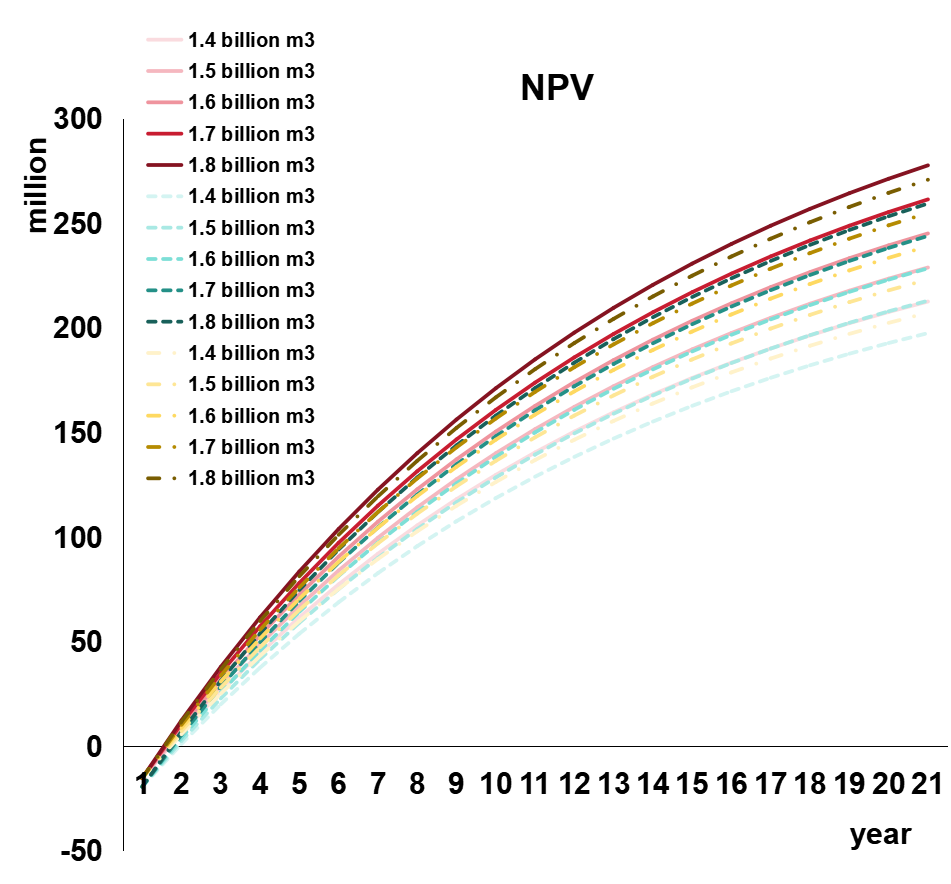

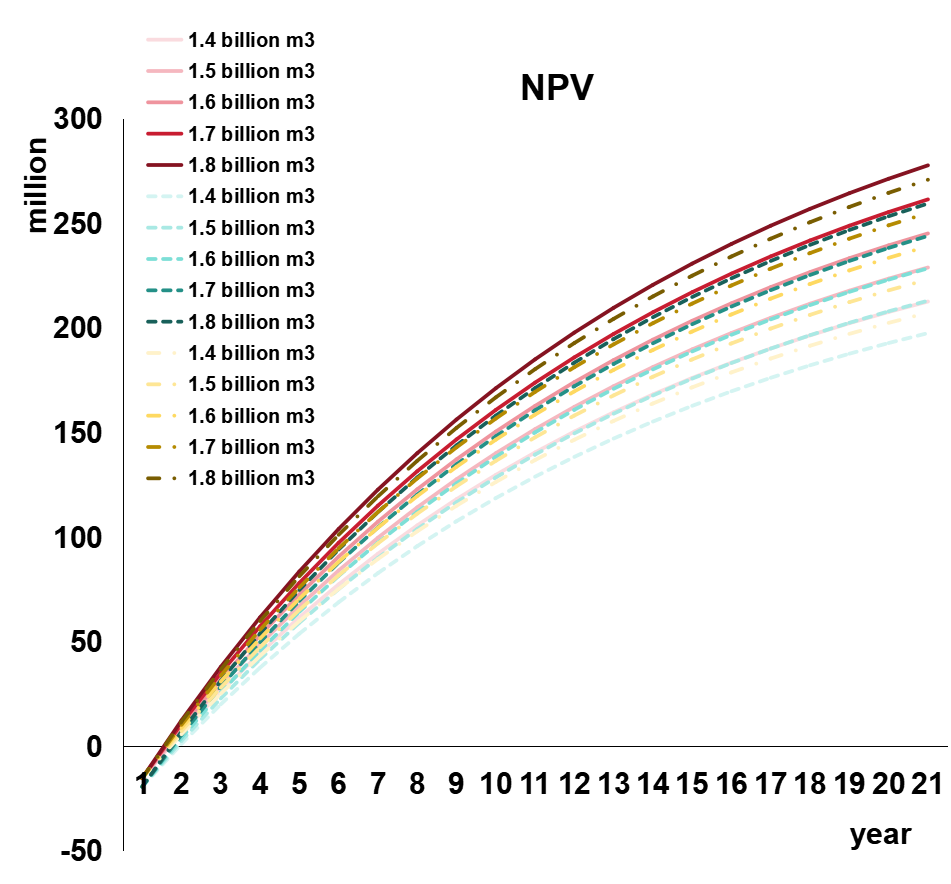

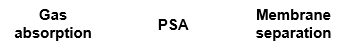


Figure S2 The NPV of 20-90% natural hydrogen produced with different separation technologies and

volumes

## Parameters of payback period

Data for P is obtained from CAPEX and OPEX of wells drilling and separation techniques mentioned above. Considering the actual production, the following assumptions are made in calculating the P-value:

- A total of 120 scenarios (i.e. 500m well depth, one off-gas disposal well) are considered. as the volume and purity of hydrogen,the number of producer wells and three separation techniques are treated as variables.
- The number of producer wells and hydrogen purity are aligned with a baseline scenario of 90% hydrogen purity and 20 production wells, and for 80% to 20% hydrogen the number of wells were 30, 40, 50, 60, 70, 80 and 90, respectively.

# S6: Unit costs of natural hydrogen extraction for various hydrogen volumes, purity and separation techniques

In actual commercial mining, unit cost is a significant indicator, and the smallest unit cost is considered for various initial scenarios. Data for unit cost is obtained from Mivechian A (*6*) and Maria Nordio (*7*), using capital investment(CAPEX), operational cost(OPEX) and annual product hydrogen volume. Considering the actual production scenario differences, we make the following assumptions:

- The utilization of equipment for hydrogen purification does not necessitate full power operation, meaning that an increase in annual hydrogen production does not result in a proportional increase in CAPEX. Furthermore, OPEX is subject to a multiple equal to the variation in annual product hydrogen volume.
- The cost values were discounted according to the initial hydrogen purity and the product hydrogen purity after purification within the reference, as shown in Eq. (4).

${unit cost}^{*}=\frac{P_{\mathrm{product}H_{2}}-P_{\mathrm{initial}H_{2}}}{P_{\mathrm{product}H_{2}}-P_{\mathrm{natural}H_{2}}}\times$unit cost Eq. (4)

where the unit cost with * is the discounted value we get.


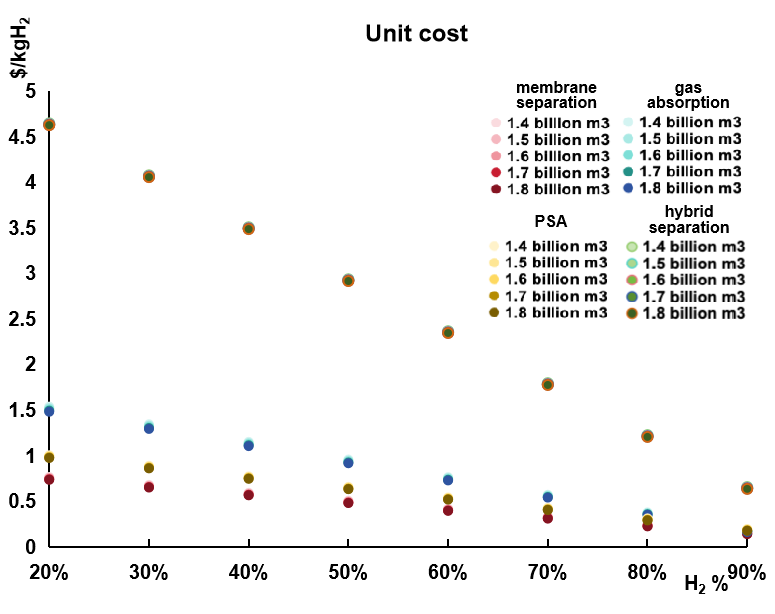


Figure S3 The minimum unit costs of nature hydrogen extraction with different hydrogen volume, purity and separation techniques

References:

1. V. Zgonnik, The occurrence and geoscience of natural hydrogen: A comprehensive review. (2020). https://doi.org/10.1016/j.earscirev.2020.103140.

2. A. R. Brandt, Greenhouse gas intensity of natural hydrogen produced from subsurface geologic accumulations. *Joule* **7** (2023).

3. J. Guélard, V. Beaumont, V. Rouchon, F. Guyot, D. Pillot, D. Jézéquel, M. Ader, K. D. Newell, E. Deville, Natural H2 in Kansas: Deep or shallow origin? *Geochemistry, Geophys. Geosystems* **18** (2017).

4. A. Prinzhofer, C. S. Tahara Cissé, A. B. Diallo, Discovery of a large accumulation of natural hydrogen in Bourakebougou (Mali). *Int. J. Hydrogen Energy* **43** (2018).

5. M. Z. Lukawski, B. J. Anderson, C. Augustine, L. E. Capuano, K. F. Beckers, B. Livesay, J. W. Tester, Cost analysis of oil, gas, and geothermal well drilling. (2014). https://doi.org/10.1016/j.petrol.2014.03.012.

6. A. Mivechian, M. Pakizeh, Hydrogen recovery from Tehran refinery off-gas using pressure swing adsorption, gas absorption and membrane separation technologies: Simulation and economic evaluation. *Korean J. Chem. Eng.* **30** (2013).

7. M. Nordio, S. A. Wassie, M. Van Sint Annaland, D. A. Pacheco Tanaka, J. L. Viviente Sole, F. Gallucci, Techno-economic evaluation on a hybrid technology for low hydrogen concentration separation and purification from natural gas grid. *Int. J. Hydrogen Energy* **46** (2021).
